# Scale ambiguities in material recognition

**DOI:** 10.1016/j.isci.2022.103970

**Published:** 2022-02-22

**Authors:** Jacob R. Cheeseman, Roland W. Fleming, Filipp Schmidt

**Affiliations:** 1 Department of Experimental Psychology, Justus Liebig University Giessen, Otto-Behaghel-Str. 10F, 35394 Giessen, Germany; 2 Center for Mind, Brain and Behavior (CMBB), Hans-Meerwein-Strasse 6, 35032 Marburg, Germany

**Keywords:** Biological sciences, Neuroscience, Sensory neuroscience

## Abstract

Many natural materials have complex, multi-scale structures. Consequently, the inferred identity of a surface can vary with the assumed spatial scale of the scene: a plowed field seen from afar can resemble corduroy seen up close. We investigated this ‘material-scale ambiguity’ using 87 photographs of diverse materials (e.g., water, sand, stone, metal, and wood). Across two experiments, separate groups of participants (*N* = 72 adults) provided judgements of the material category depicted in each image, either with or without manipulations of apparent distance (by verbal instructions, or adding objects of familiar size). Our results demonstrate that these manipulations can cause identical images to be assigned to completely different material categories, depending on the assumed scale. Under challenging conditions, therefore, the categorization of materials is susceptible to simple manipulations of apparent distance, revealing a striking example of top-down effects in the interpretation of image features.

## Highlights

• Interpretations of ambiguous surface materials varied with assumed viewing distance
• Causal manipulation of assumed viewing distance reproduced this effect
• Canonical spatial scale can inform surface material inference under uncertainty

## Graphical abstract

**Figure:**
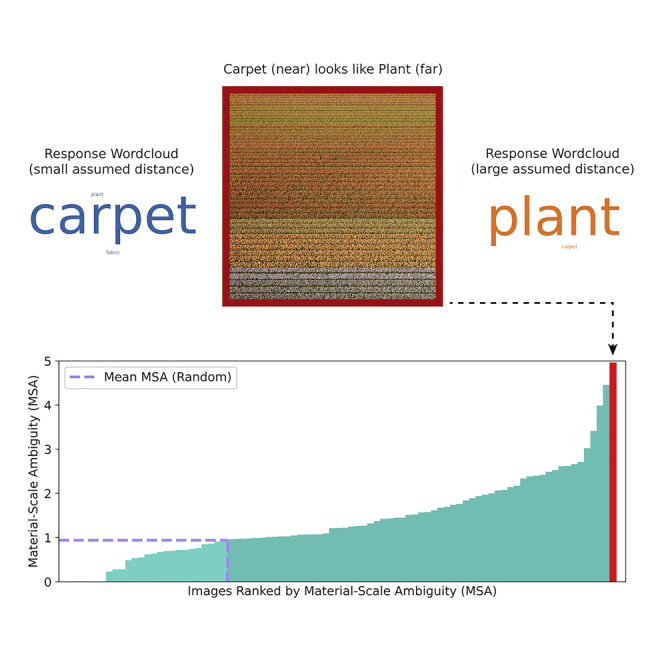

## Introduction

**Figure 1:**
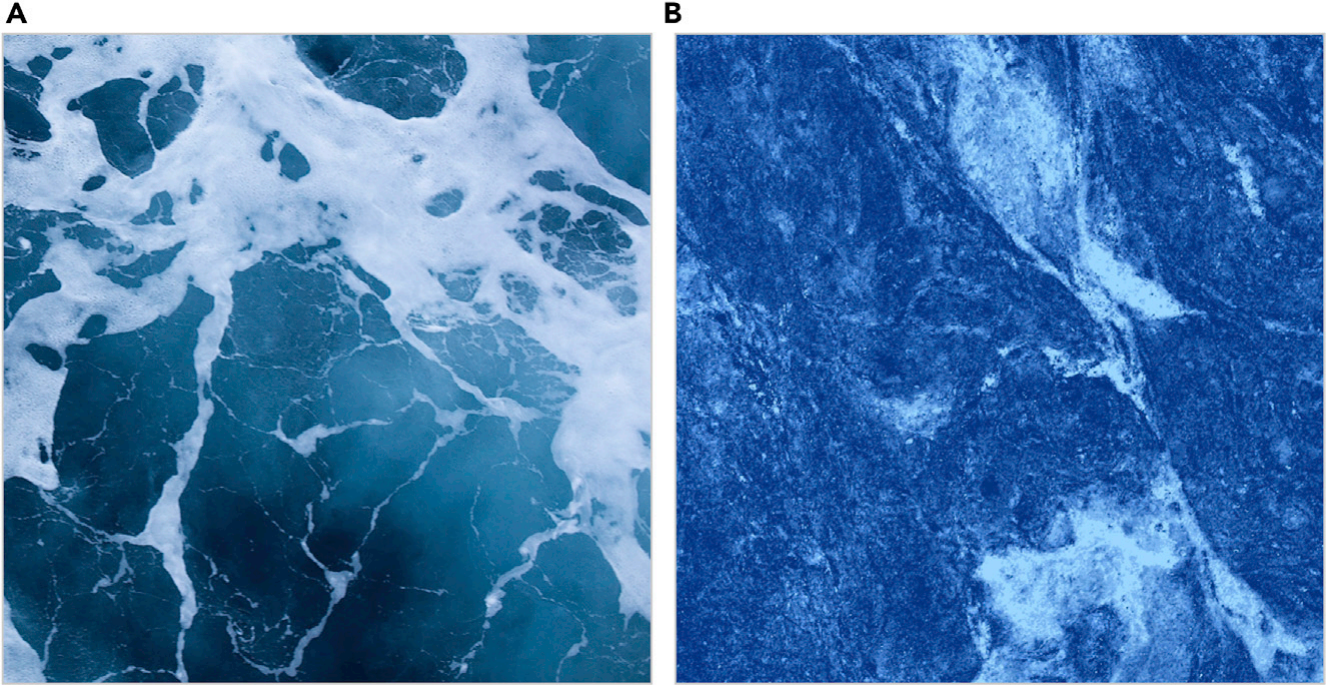
Demonstration of material and scale dependency When viewing distance is ambiguous, the material properties depicted in these photographs of (A) sea water and (B) marble are confusable. Note that the relative difference in assumed viewing distance (i.e., the ocean water is farther than the marble surface) reverses if one imagines that the labels are switched.

In most circumstances, observers can reliably and efficiently recognize the materials that surfaces are made of (Sharan et al., 2014). However, here we find that under certain viewing conditions, surfaces made of entirely different materials can produce similar image features, leading to confusions. For example, the mottled surfaces in Figure 1 are photographs of (A) sea water and (B) marble. When asked to estimate the distances in these images, we are likely to guess that the sea water is much farther from the camera than the marble surface. If we instead imagine that (B) is sea water and (A) is marble, this reverses the difference in assumed distance; that is, sea water only appears this way from a relatively large distance. This ‘material-scale ambiguity’ suggests that our knowledge of the typical appearance of materials can be used to constrain the range of plausible viewing distances that could produce these images (Fleming, 2014), much like objects of familiar size can indicate depth and distance (Hubbard et al., 1989; Konkle and Oliva, 2011, 2012a, 2012b). Conversely, if material identity and spatial scale are mutually constraining, this also raises the possibility that judgments of material identity may be influenced in a top-down way by the assumed viewing distance to objects and surfaces.

Material appearance is intimately connected to the spatial scale of geometrical and optical processes at which light interacts with surfaces (Pont and Koenderink, 2002, 2005). A given material can change in appearance dramatically depending on the viewing distance. Individual water droplets seen close-up are visibly transparent with small punctate highlights, yet when seen *en masse* from afar, droplets blur together into a white haze. Tiny scratches on the surface of brushed metal appear crisp and mirror-like when viewed through a microscope, yet the ensemble appearance at normal view distances is of a silky, anisotropic gloss (Raymond et al., 2016). We reasoned that given the complex, multi-scale nature of many materials, image features that emerge at a particular scale or viewing distance can sometimes be deceptive about material properties. For example, anyone who has touched the stems and spikelets of barley knows that they are harsh to touch, yet when seen from afar, a field of barley can have a distinctly ‘soft’ appearance. This opens the possibility of mis-categorizing materials as a function of the interpreted or assumed viewing distance.

Here, we investigated how contextual information about viewing distance influences material recognition. We did not seek to test the claim that *all* images of materials suffer from material-scale ambiguities, but rather the more restricted claim that there are certain categories of materials that are confusable when imaged from different viewing distances—and thus that certain images can exhibit this effect. We explicitly set out to find such images and investigate the patterns of confusions they cause. If the same image can be interpreted as completely different material categories depending on the viewer's assumptions about viewing distance, this has important implications for theories of visual categorization, which typically assume a mapping between particular image features and material categories (Bell et al., 2015; Fleming et al., 2013; Sharan et al., 2013).

## Results

### Experiment 1: unbiased judgements of distance and material

**Figure 2:**
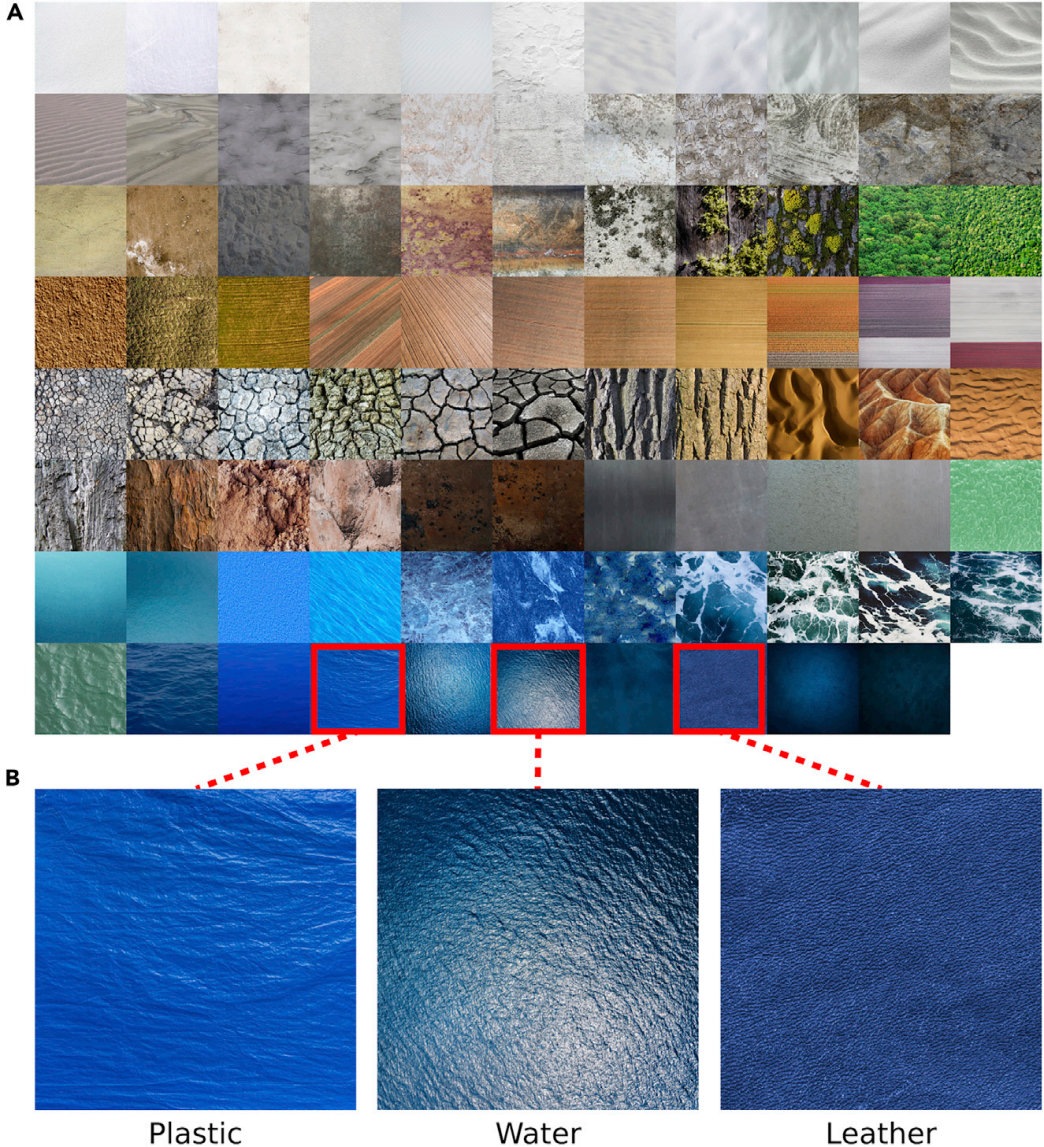
Stimuli for Experiments 1 and 2 The full set of 87 images (A) is arranged by approximate visual similarity. Similar image features (e.g., color, texture) can arise from different material categories (B), especially when spatial scale is ambiguous.

We first sought to measure baseline judgements of distance and material for our stimulus set in the absence of explicit distance or scale information. Participants were presented with a series of potentially ambiguous photographs (Figure 2A; see STAR Methods: Stimuli) and asked to estimate the distance between the camera and depicted surface plane, and to identify the material category. The unbiased responses from this experiment served as a baseline of comparison with Experiment 2, in which judgements of distance and material were manipulated with contextual information.

Our first main finding is that, overall, participants were quite good at identifying the correct categories of materials. All participants achieved categorization accuracies (*Mdn* = 33%) that exceeded chance-level accuracy (4%). According to the 95% confidence interval (CI) obtained from a one sample binomial test, the probability that the observed median categorization accuracy exceeds chance-level is between 96% and 100%, *p* < 0.001, *g* = 0.29. In addition, there was a strong rank correlation between the frequency of common terms represented in the free-response and multiple-choice data (*r*_*s*_ = 0.76, *p* < 0.001; see Free-Response Transformations in the STAR Methods for details). These findings show that although the images were selected for their ambiguity, participants' responses were systematic and consistent across tasks. Thus, deviations from ground truth were likely purposeful. We next sought to answer whether such errors were systematically related to the assumed scale (or distance) of the scene.

To assess this, we divided the multiple-choice responses for each image into two *distance groups*: (i) responses from participants who selected distance units indicating a relatively small scale (*near group*: micrometer, millimeter, or centimeter), and (ii) responses from participants who selected distance units that indicated a relatively large scale (*far group*: meter or kilometer). Material category confusions can occur in two directions (near → far vs. far → near). Some confusions between categories are likely to be distance-independent simply because the materials are similar in appearance (e.g., confusing stone and concrete irrespective of distance), and will therefore tend to be symmetrical in each direction. Instead, we focus on *asymmetrical* patterns of confusion that reflect a *distance-dependent* material ambiguity. That is, are there images that are assigned to one material (e.g., bark) when seen as close-up, which are assigned to another material (e.g., stone) when seen as far away, but not vice versa?

**Figure 3:**
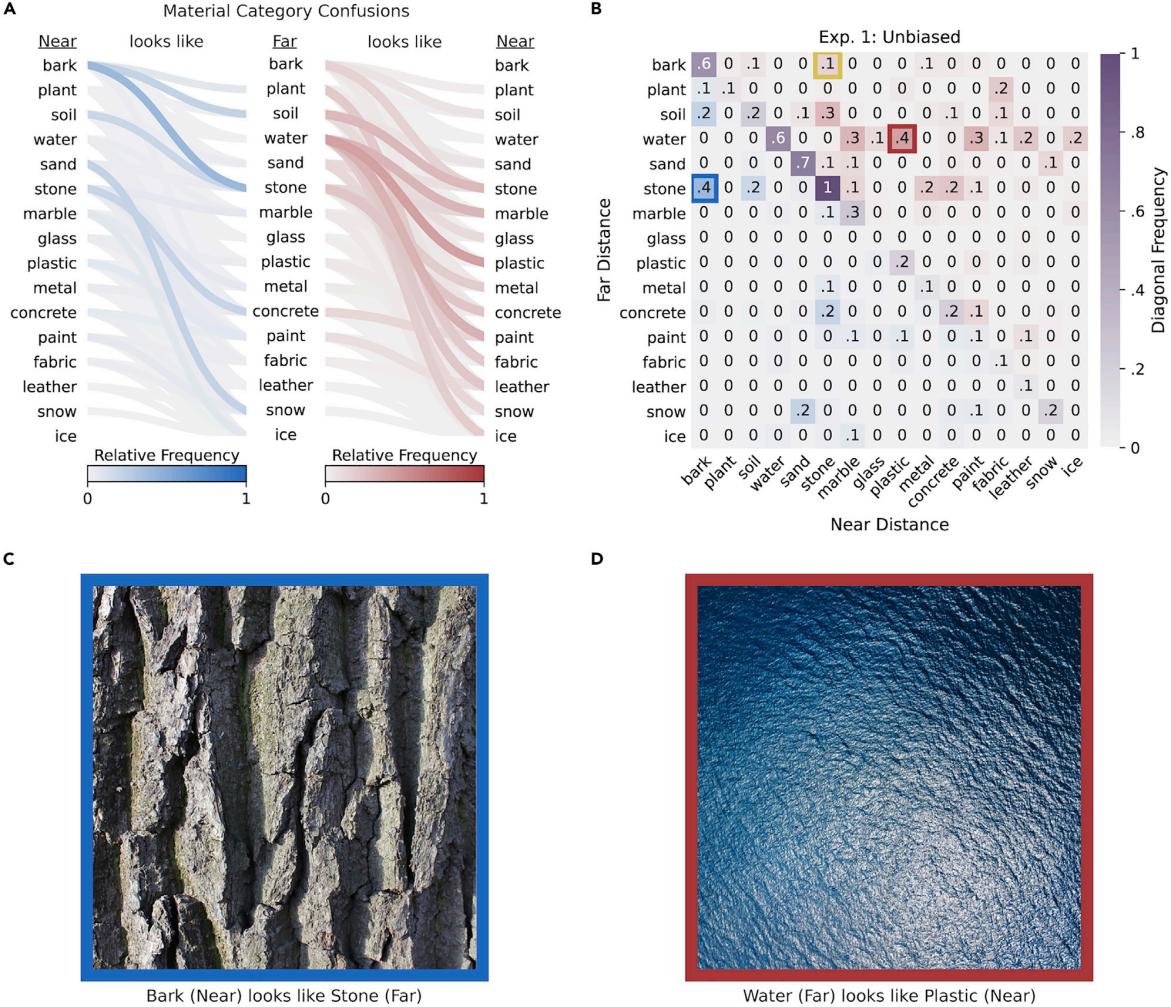
Material category confusions exhibiting material-scale ambiguity The relative frequency of confusions between pairs of categories is indicated by shaded connecting lines in (A). Categories selected with smaller distance units (micrometer, millimeter, or centimeter) are defined as *near*, whereas categories selected with larger distance units (meter or kilometer) are defined as *far*. Confusions in the near → far direction (blue-shaded lines) differ systematically from confusions in the far → near direction (red-shaded lines). The same asymmetry can be seen when (normalized) relative frequencies are plotted in a matrix format (B), with distance-dependent responses on separate axes. Category pairs above and below the negative diagonal indicate the relative frequency of distance-dependent confusions; distance-independent responses are shown on the diagonal. The top-ranked images for the most frequent near → far confusion (C) and the most frequent far → near confusion (D) illustrate different material-scale ambiguities

The relative frequency of directional category confusions is indicated by shaded lines in Figure 3A. The observed asymmetry between the pattern of blue lines (near → far confusions) and red lines (far → near confusions) illustrates that material category confusions vary systematically with assumed viewing distance. For clarity, cases where the two distance groups selected the same category are omitted, and only categories corresponding to ground truth image labels are shown (for details, see Relative Frequencies in the STAR Methods).

Figure 3B displays the same relative frequencies (here normalized between 0 and 1) plotted as cells in a matrix, with responses of the near and far groups represented on separate axes. Category confusions that occur in the near → far direction are located in the lower triangle of the matrix (blue-shaded cells), and confusions in the far → near direction are located in the upper triangle (red-shaded cells). Instances where the two distance groups selected the same category are plotted on the diagonal of the matrix (purple-shaded cells). Directional asymmetries can be compared by examining category pairs on opposite sides of the diagonal. For example, we find that surfaces identified as *bark* at smaller apparent distances tend to be confused with *stone* at larger apparent distances (frequency = 0.4; blue square in Figure 3B). However, the reverse is not true: when a surface is seen as *bark* at larger apparent distances, it is less often confused with *stone* at smaller apparent distances (frequency = 0.1; yellow square in Figure 3B). Figure 3C shows one such image, and for comparison Figure 3D shows an image with a distance-dependent ambiguity in the opposite direction (water seen from far looks like plastic seen from near).

**Figure 4:**
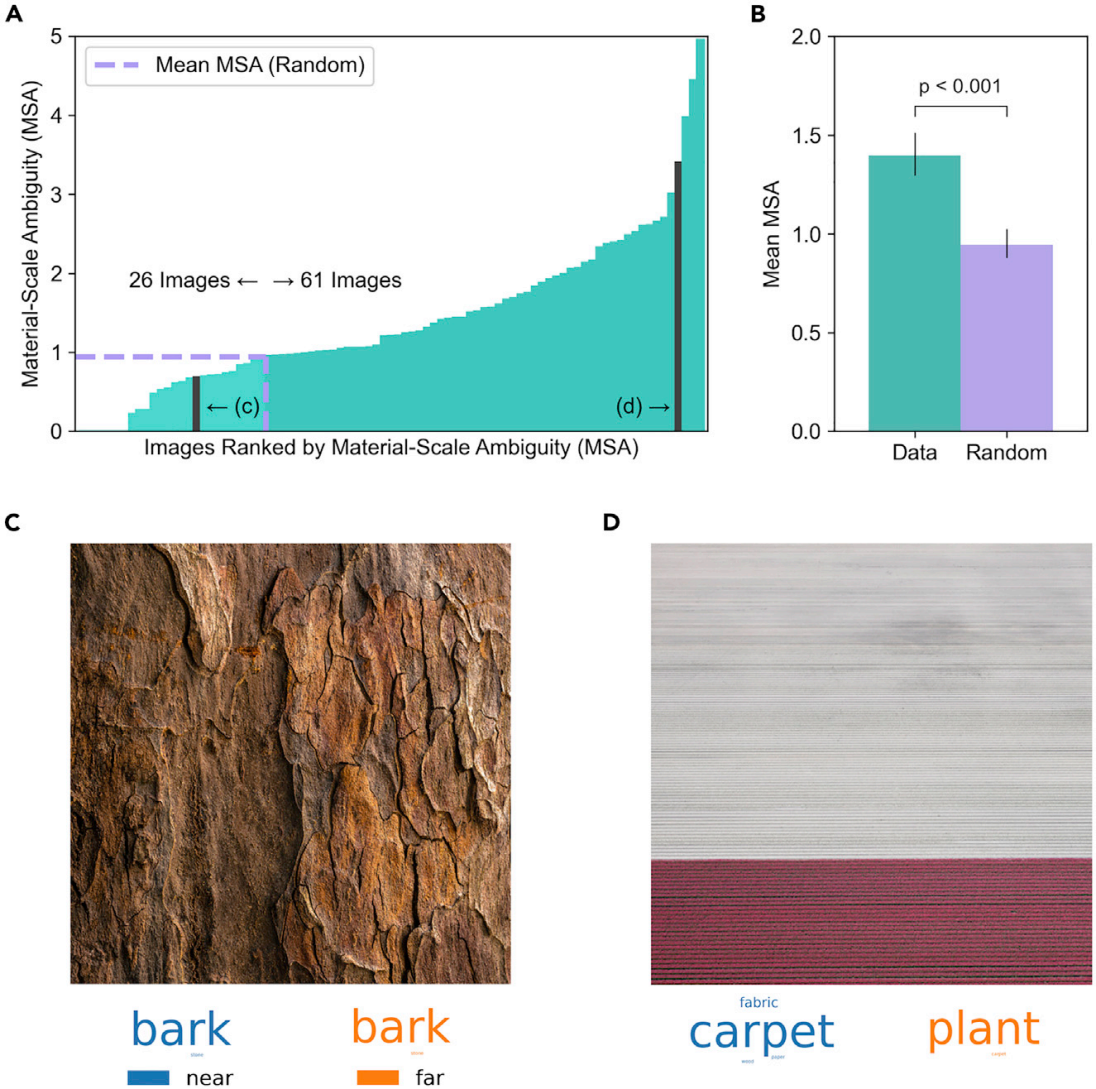
Material-Scale Ambiguity (MSA) calculated for individual images Images can be ranked by MSA (A) along with the mean MSA resulting from random permutation of distance units drawn in lilac. The mean MSA across images (B) for the original responses (teal) and random permutation of distance units (lilac). A Wilcoxon signed-rank test indicated that the observed difference in MSA is significant. Error bars represent 95% CIs. An image with low MSA (C) is reliably seen as one material (bark) regardless of assumed distance. Word clouds of responses (size weighted by frequency) associated with different distance assumptions are shown below. An image with high MSA (D) is reliably seen as different materials (carpet vs. plant) depending on the assumed distance (far vs. near).

The matrix representation of category confusions also provided a simple way to quantify material-scale ambiguity for these images. We did this by calculating the Root Mean Squared Error (RMSE) between corresponding frequencies in the lower and upper triangles of the matrix (e.g., blue cells vs. red cells in Figure 3B, excluding the diagonal). This metric, which we call the *Material-Scale Ambiguity* (MSA), represents the amount of directional asymmetry in category confusions. MSA can also be calculated for individual images, which allows us to identify the subset of images that were particularly effective in producing distance-dependent category confusions. MSA for individual images was then compared with asymmetries that occur by chance, by calculating the mean MSA across 1000 random permutations of the distance units associated with each response (i.e., category confusions are shuffled in each direction). Figure 4A shows images ranked by MSA, with the corresponding chance-level MSA drawn in lilac. Sixty-one (out of 87) images produced MSA values above chance. Figure 4B shows that the mean MSA across all images (teal; *M* = 1.4, 95% CI [1.3, 1.51]) was significantly greater than chance-level MSA (lilac; *M* = 0.95, 95% CI [0.88, 1.02]; Wilcoxon *T* = 850, *p* < 0.001, *d* = 0.52). This result indicates that, as a whole, the image set produces systematic distance-dependent category confusions, but also that there is a subset of images that are particularly ambiguous. For example, the image shown in Figure 4C was reliably seen as *bark* regardless of apparent distance, whereas the image shown in Figure 4D produced confusions between *plant* (seen from a relatively large distance) and *carpet* (seen from a relatively small distance). The material categories associated with relatively small or large assumed distances are shown in word clouds below each image, where category frequency is indicated by word size.

Taken together, the results of this experiment show that, without contextual information to specify viewing distance, certain images exhibit a striking distance-dependent ambiguity about the category of material. The material category labels assigned to these images covary with whether the surfaces are interpreted as near to or far from the camera. To test whether the assumed viewing distance plays a *causal* role in driving this effect, we next sought to directly manipulate assumed viewing distance.

### Experiment 2: biased judgements of distance and material

**Figure 5:**
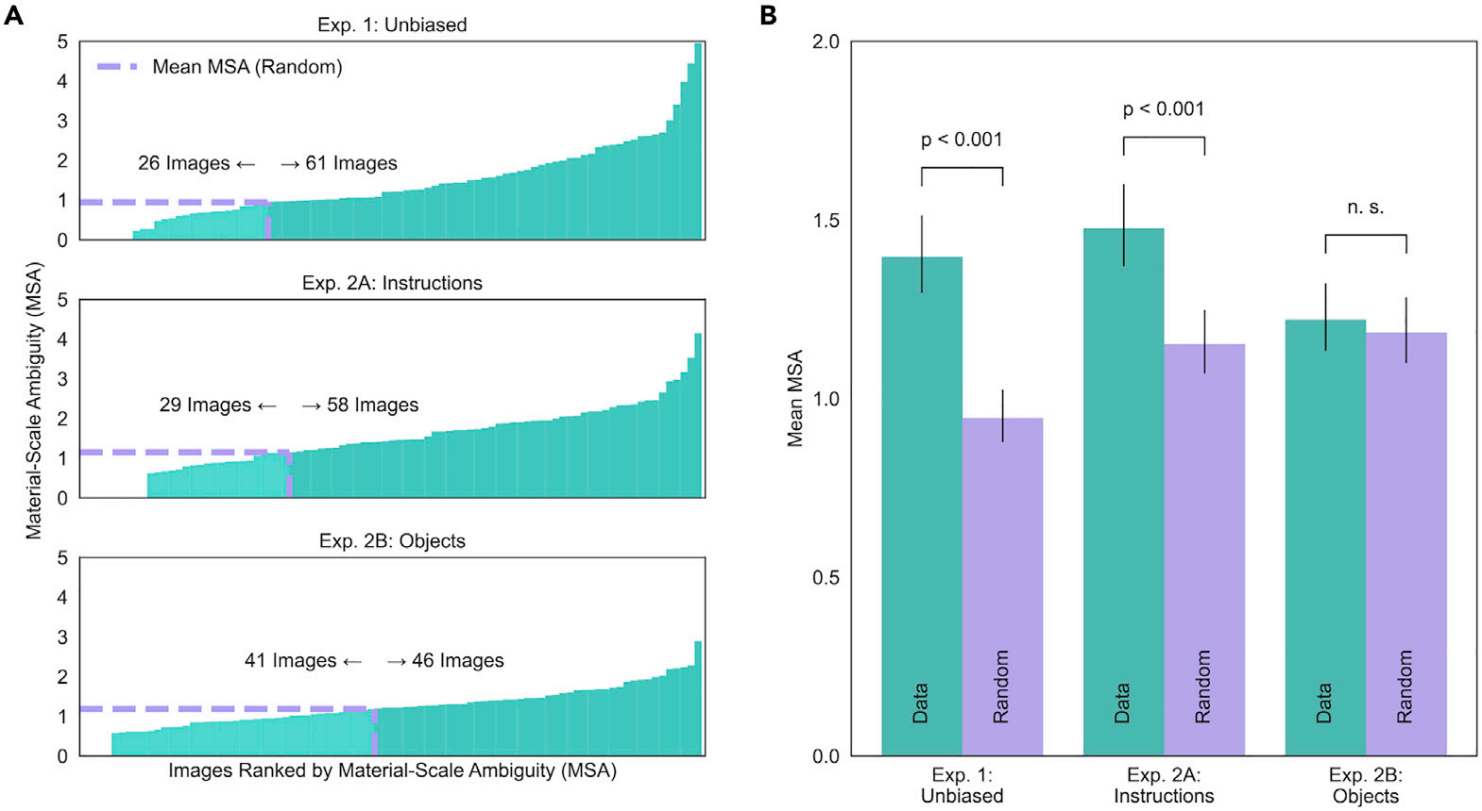
Material-Scale Ambiguity (MSA) calculated separately for each experiment (A) Images ranked by MSA. The mean MSA resulting from random permutation of distance units is drawn in orange. (B) Mean MSA across images for the original responses is shown in blue, whereas mean MSA obtained from random permutation of distance units (Experiment 1) or group membership (Experiment 2) is shown in orange. The observed difference in MSA is significant for Experiments 1 (unbiased) and 2A (instructions). Error bars represent 95% CIs

To test whether manipulating assumed viewing distance influences judgements of material, we presented the same stimuli to four additional groups of naive participants. Assumed viewing distance was manipulated by instructing each group to imagine a small or large distance to the surface plane (Experiment 2A), or by presenting modified images that featured two different familiar objects to indicate different spatial scales (Experiment 2B). Material-Scale Ambiguity (MSA) was calculated separately for Experiment 2A and Experiment 2B. To test whether the observed asymmetries in category confusions were statistically significant, we compared them to chance-level MSA calculated from 1000 random permutations of group membership in each experiment. Images ranked by MSA for all experiments are shown in Figure 5A, along with chance-level MSA in lilac. Figure 5B shows the mean MSA across images for the original responses (teal) and chance-level MSA (lilac) separately for all experiments.

In Experiment 2A, MSA values for the original responses (*M* = 1.48, 95% CI [1.37, 1.6]) were significantly greater than chance (*M* = 1.15, 95% CI [1.07, 1.25]; Wilcoxon *T* = 799, *p* < 0.001, *d* = 0.49), and comparable to the unbiased judgements of material and distance in Experiment 1. Although familiar objects produced above-chance MSA in 46 images (Experiment 2B), this manipulation was less effective overall (Wilcoxon *T* = 1812, *p* = 0.80, *d* = 0.07). Interestingly, confidence ratings in Experiment 2B (*M* = 4.8, *SD* = 0.71) were significantly lower than those in Experiment 2A (*M* = 4.98, *SD* = 0.5; Mann-Whitney *U* = 192.5, *p* = 0.025, *d* = 0.25). It is also noteworthy that in general, the same images produced high MSA across experiments. MSA values in Experiment 1 are significantly correlated in rank with those obtained in Experiment 2A (*r*_*s*_ = 0.43, *p* < 0.001) and Experiment 2B (*r*_*s*_ = 0.28, *p* = 0.009); the rank correlation between Experiment 2A and 2B was not significant. These results suggest that, compared to instructions, the effectiveness of familiar objects is more dependent on whether the objects appear plausible within a given scene (e.g., whether the lighting of the virtual objects was congruent with the lighting of the real scene).

**Figure 6:**
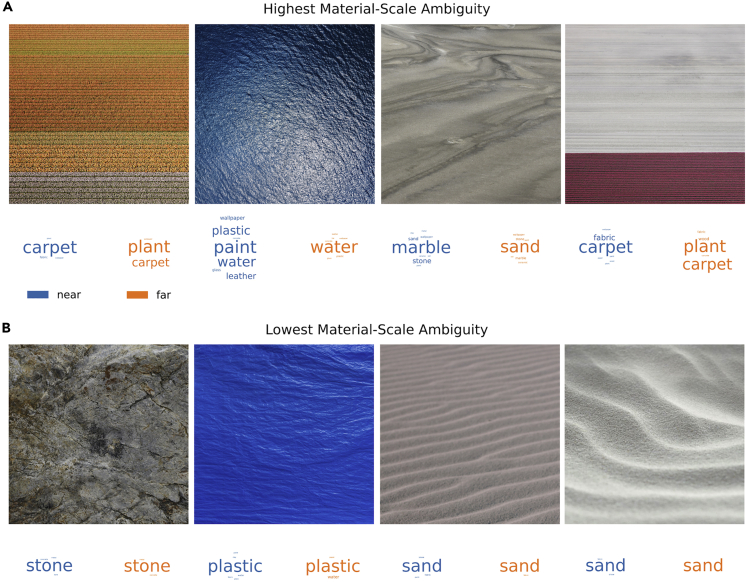
Images from our set with the highest and lowest MSA across experiments Word clouds below each image show the distinct material categories (size weighted by frequency) selected by participants who assume relatively small or large distances to the surface plane (blue and orange data, respectively).

Ranking our images by the mean MSA across experiments provides the clearest summary of our findings (Figure 6). Images with low MSA (bottom row) are immune to manipulations of viewing distance. Yet, when MSA is high (top row), the assumed distance between the camera and the surface plane determines which categories are selected.

## Discussion

Surface material appearance is characterized by complex physical and optical structures at multiple spatial scales. As materials can have strikingly different appearances at different viewing distances, we reasoned that for certain images, the appearance of a material at one distance could potentially resemble the appearance of another material at a different distance, leading to different category assignments. Here, we have established that such images exist and shown how the assumed view distance can radically alter how they are interpreted.

In Experiment 1, we found spontaneous confusions about material category co-occurring with the assumed viewing distance. In Experiment 2, we showed that both instructions and the insertion of familiar objects indicating different physical scales can alter material classification in a subset of images. An important caveat is that our conclusions are necessarily dependent on the images in our stimulus set. Our goal was to identify specific images that are susceptible to such manipulations, rather than to draw conclusions about the frequency of such ambiguities across all natural images. Although we rarely make such confusions about material categories in everyday life, the very existence of such images demonstrates a substantial top-down effect in visual recognition, in which context can radically alter the interpretation of identical images.

Our finding that distance assumptions can bias material categorization supports the notion that materials have *canonical scales* that constrain diagnostic image cues. Much as certain viewpoints and sizes of objects can be considered canonical (Konkle and Oliva, 2011; Palmer et al., 1981), image cues associated with particular material categories may be more likely to arise at *typical* viewing distances (Fleming, 2014). Conversely, when a material is viewed from an *atypical* distance (e.g., in macro or aerial photography; see De Giuli, 2018), the cues may resemble other materials, resulting in material-scale ambiguities. To date, theories of material perception and appearance have tended to ignore their scale-specificity; future work on material recognition should consider how material appearance and relevant image features vary with distance.

### Image cues and material-scale ambiguity

Which image characteristics cause material-scale ambiguity, rather than the general kind of ambiguity that is unrelated to apparent scale? Are there any features that predict the ambiguity across classes, or is the phenomenon driven by different cues for different materials? The images in Figure 6 hint at some compositional elements that might contribute. When the camera is (approximately) perpendicular to the surface plane, this limits depth cues (e.g., texture and optical blur gradients; Cutting and Vishton, 1995; Hafri et al., 2021; Sedgwick, 2005; Vishwanath, 2010), presumably increasing their ambiguity. The absence of visible object boundaries likely also plays an important role, although many such unbounded texture images are unambiguous (Fleming et al., 2013; Wiebel et al., 2013). Moreover, if an image contains scale-invariant structure (e.g., fractal or self-similar texture; see Brachmann and Redies, 2017), such image features provide little information about viewing distance, whereas image features that do vary with viewing distance may be more diagnostic of certain material categories.

If there are certain cues whose presence or absence tends to increase material-scale ambiguity, then it should be possible to predict the degree of ambiguity across material classes based on these features. In a first attempt to test this, we identified five appearance characteristics that could plausibly be relevant to material-scale ambiguity (atmospheric blue tint, blurriness, direct lighting, surface gloss, and surface slant) and obtained human ratings for all images in our dataset, along with one image metric (self-similarity). However, a classifier based on these feature values failed to accurately classify high-MSA vs. low-MSA images, although we did find that slanted surfaces and natural materials were associated with larger apparent distances (for details, see STAR Methods: Exploratory analyses). These results suggest that the image statistics that would predict MSA for a given pair of categories (e.g., bark and stone) likely differ from those needed for another pair of categories (e.g., water and plastic).

Considering the great diversity of appearances associated with different material classes, it is unlikely that a single image characteristic exists that can determine whether a given image exhibits material-scale ambiguity across all possible category confusions. Yet, for a given material category, there may be certain cues that are particularly important. For example, Sawayama et al., 2017 have shown that participants can judge the fineness of fibrous textures (like human hair), even when individual texture elements are smaller than the resolution of the imaging system. The main cue driving the super-resolution judgments in their displays was contrast: lower contrast patterns appeared to contain finer elements because of averaging of texture elements within each pixel. If the visual system relies on such inferences beyond the image data to infer specific material properties, it is necessarily open to scale-related ambiguities. Our results demonstrate that material recognition can depend on the ‘beholder’s share’ rather than image statistics *per* *se* (Gombrich, 1961).

### Limitations of the study

**Figure 7:**
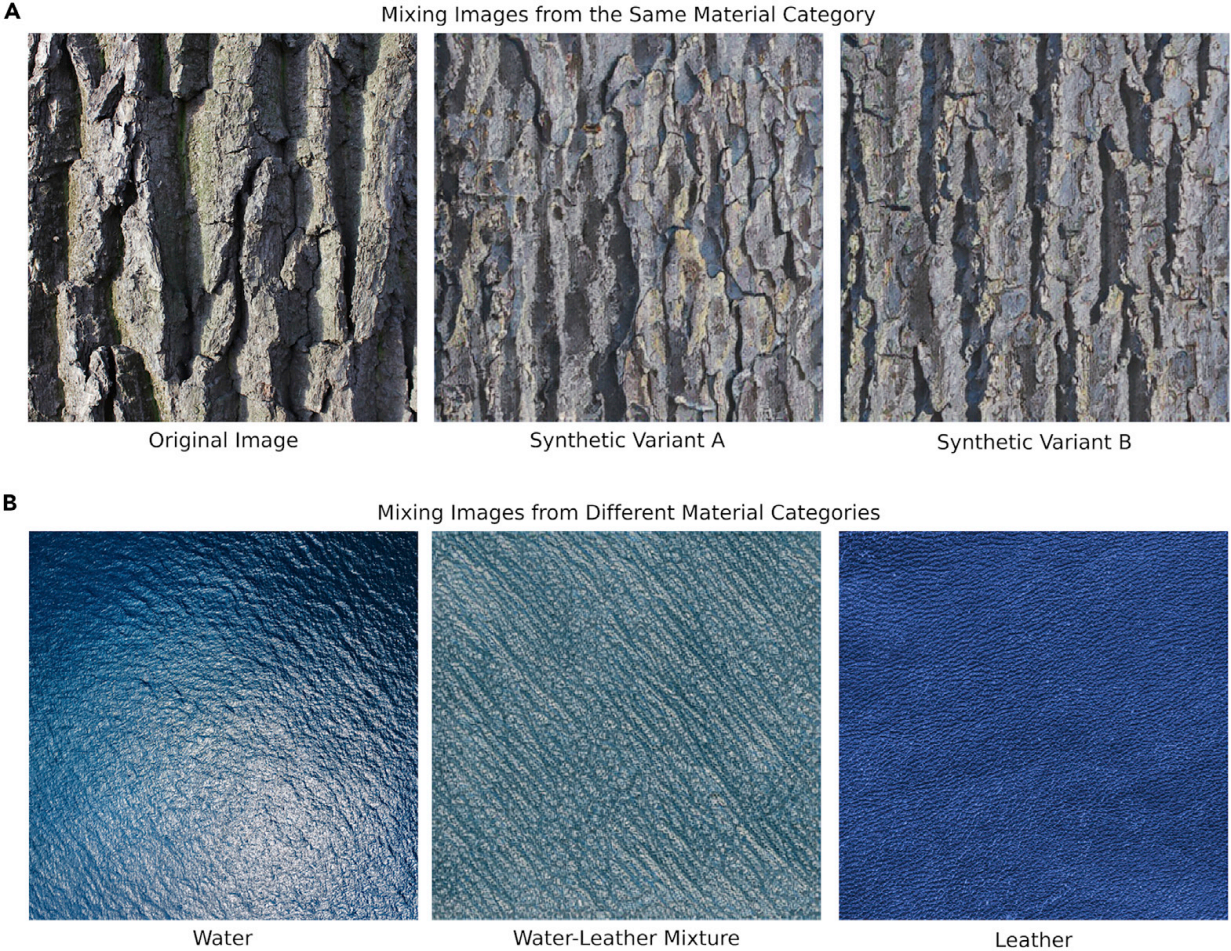
Material ambiguity might be created with the aid of deep neural networks (A) An original image from our set can be modified to mimic the image statistics of other exemplars from the same material category. (B) Original images from different material categories (water and leather) can be used to create a synthetic image that inherits the statistics of both images. These techniques could be used to aid the discovery or invention of images that possess specific kinds of material ambiguity

The conclusions we can draw from this study are necessarily limited to specific images that exhibit material-scale ambiguity. Further investigation of this effect—and how it relates to material perception in general—will require innovations that lower the difficulty of creating or discovering such images. One approach to probing the characteristics of such images is to blend the statistics of different images that produce the same class confusions. For example, Figure 7A displays an image of *bark* that was often confused with *stone*, together with two synthetic ‘lookalikes’ generated by a deep convolutional neural network used for image style transfer (Gatys et al., 2016). In this example, the ‘styles’ are other images of *bark* that were often confused with *stone* (i.e., the ground truth category is the same in the style and transfer images). A variation of this approach could involve mixing images that differ in ground truth category, but which produce the same kind of material-scale ambiguity (e.g., the images of water and leather in Figure 7B). An intriguing alternative approach would be to ask artists to create ambiguous images and analyze the techniques they use to depict material appearance (Di Cicco et al., 2021; Lin et al., 2021). Such methods may make it possible to identify key features that alter perceived scale or material appearance.

## STAR★Methods

### Key resources table

**Table:** 

REAGENT or RESOURCE	SOURCE	IDENTIFIER
**Deposited Data**		

Experimental stimuli, behavioral data, and analysis code	Open Science Foundation	https://doi.org/10.17605/OSF.IO/UKQ95

**Software and Algorithms**		

Qualtrics Survey Platform v02.2019	https://www.qualtrics.com	RRID:SCR_016728
Python v3.7.6	https://www.python.org/downloads/release/python-376/	RRID:SCR_008394
Jupyter Notebook v6.4.2	https://github.com/jupyter/notebook/tree/v6.4.2	RRID:SCR_018413
NumPy v1.20.3	https://github.com/numpy/numpy/tree/v1.20.3	RRID:SCR_008633
pandas v1.3.1	https://github.com/pandas-dev/pandas/tree/v1.3.1	RRID:SCR_018214
SciPy v1.6.2	https://github.com/scipy/scipy/tree/v1.6.2	RRID:SCR_008058
Scikit-learn v0.24.2	https://github.com/scikit-learn/scikit-learn/tree/0.24.2	RRID:SCR_002577
matplotlib v3.4.2	https://github.com/matplotlib/matplotlib/tree/v3.4.2	RRID:SCR_008624
seaborn v0.11.1	https://github.com/mwaskom/seaborn/tree/v0.11.1	RRID:SCR_018132
Statannotations v0.4.3	https://github.com/trevismd/statannotations	N/A
spacy v3.1.2	https://github.com/explosion/spaCy/tree/v3.1.2	N/A
tensorflow v2.0.0	https://github.com/tensorflow/tensorflow/tree/v2.0.0	RRID:SCR_016345
tensorflow_hub v0.10.0	https://github.com/tensorflow/hub/tree/v0.10.0	N/A
R v4.0.2	https://cloud.r-project.org/bin/macosx/base/	RRID:SCR_001905
rpy2 v3.4.5	https://rpy2.github.io/doc/v3.4.x/html/index.html	N/A
MATLAB R2021a	https://www.mathworks.com/downloads/	RRID:SCR_001622
MATLAB Engine API for Python	https://www.mathworks.com/help/matlab/matlab_external/install-the-matlab-engine-for-python.html	N/A

**Other**		

Eizo ColorEdge CG277 LCD Monitor	https://www.eizo.com/products/coloredge/cg277/	N/A

### Resource availability

#### Lead contact

Further information and requests for resources should be directed to and will be fulfilled by the lead contact, Jacob R. Cheeseman (jacob.cheeseman@psychol.uni-giessen.de).

#### Materials availability

All materials are included in the text and Supplemental information.

### Experimental model and subject details

In Experiment 1, a convenience sample of 24 students (14 women, 10 men; *M*_*age*_ = 24.3 years, *SD*_*age*_ = 3.3 years) participated. In Experiment 2, four groups of 12 students (48 total) participated. Group 1 (9 women, 3 men; *M*_*age*_ = 24.5 years, *SD*_*age*_ = 3.01 years) and Group 2 (9 women, 3 men; *M*_*age*_ = 22.5 years, *SD*_*age*_ = 3.64 years) participated in Experiment 2A. Group 3 (4 males and 8 females; *M*_*age*_ = 24.5 years, *SD*_*age*_ = 4.01 years) and Group 4 (4 males and 8 females; *M*_*age*_ = 22.83 years, *SD*_*age*_ = 2.37 years) participated in Experiment 2B. Participants were paid an hourly rate of 8€. All experimental procedures were approved by the Justus Liebig University Giessen Psychology Department Ethics Board and conformed with the guidelines of the American Psychological Association (Version 2017) and the Declaration of Helsinki (Version 2013, excluding pre-registration). Informed consent was obtained from all subjects.

### Method details

#### Sample size

Only one previous study (Wiebel et al., 2013; *N* = 18) has estimated material categorization accuracy under conditions similar to the current study, that is, with unlimited stimulus presentation time and no instruction to respond rapidly. All of the participants in that study achieved categorization accuracies (*Mdn* = 87%) that exceeded chance-level accuracy (25%). A one sample binomial test is needed to determine whether the set of observed values, which are not normally distributed, are significantly different from a fixed value. According to the 95% confidence interval (CI) obtained from a one sample binomial test, the probability that the observed median categorization accuracy exceeds chance-level is between 85% and 100%, *p* < 0.001, *g* = 0.62. Although this indicates that material categorization is quite robust under normal conditions, given that our stimuli were selected for ambiguity, a larger sample (*N* = 24) was preferred for Experiment 1. This sample size was also used for Experiments 2A and 2B.

#### Stimuli

Potentially ambiguous photographs were collected from various internet sources. The selection criteria were as follows: (i) they did not contain objects, but clearly depicted a surface or scene, (ii) they were determined by the experimenter to be potentially ambiguous in the sense that at least two distinct material categories could describe the surface or scene, and (iii) these material categories could be associated with different spatial scales. Three observers (including two authors) screened hundreds of images on the internet and selected those that met the criteria, resulting in 87 images (Figure 2A). Ground truth category labels and copyright information for these images are listed in the Supplemental information (Figure S1 and Table S1). Ground truth category labels for these images were determined by the original publisher. Images for which print copyright permissions could not be obtained are here replaced by synthetic textures derived from the originals (see Risser, 2020). All images were rescaled to 600 × 600 pixels.

The same set of images presented in Experiment 1 was shown to each group of participants in Experiment 2A. For Experiment 2B, we created two sets of images from the original set, each of which featured a large or small familiar object (3D models of an airplane or a hornet) that provided contextual information about the spatial scale of the scene. The objects were digitally inserted into the images with 3D modelling software that simulated lighting direction and cast shadows. The 3D models (“Boeing 787 8(1)” by turbosquid.com/companion_3d, published under Editorial Use license; “Hornet” by blendswap.com/Misfit410, published under CC-BY license), were inserted into the original images using Blender 2.79 (Stichting Blender Foundation, Amsterdam, NL). In these virtual scenes, the original images were arranged parallel to the ground plane, while the objects were positioned slightly above. The camera was positioned perpendicular to the ground plane with a point light source slightly offset from the center of the ground plane, casting a shadow of the object onto the ground plane image.

#### Equipment

The experiment was controlled by a Dell Precision T3500 desktop computer running Windows 10 and Qualtrics software (v02.2019). Stimulus images were displayed using an Eizo ColorEdge CG277 self-calibrating LCD monitor (68.4 cm diagonal; 2560 × 1440 resolution). Participants were seated in a dark room approximately 50 cm from the monitor, which displayed the images against a uniform grey background. At this viewing distance, the 600 × 600 pixel images subtended approximately 16 degrees of visual angle.

#### Procedure

On each trial in Experiment 1, participants viewed a single, randomly-selected image and judged the depicted material, followed by the distance between the camera and surface plane. Different kinds of responses were collected in separate blocks of trials. In the first block (*free-response*), participants described the surface material as precisely as possible by typing a written response. In the second block (*multiple-choice*), participants identified surface materials by selecting one category from 26 options (see Multiple-Choice Instructions in the Supplemental information). Many of these 26 material categories were featured in previous studies of material perception with natural images (Bell et al., 2015; Sharan et al., 2014) while others were added by the authors to accommodate images that were not adequately described by any of the preexisting categories. Participants also provided a confidence rating (from 1 to 7) for judgements of material. In the third block (*distance estimation*), participants estimated the distance between the camera and surface plane by selecting an appropriate unit of measurement (micrometers, millimeters, centimeters, meters, or kilometers) and metric value (e.g., 8 cm, or 1 km). Similarly, in Experiment 2, participants identified the surface material (free-response and multiple-choice, in separate blocks of trials) and indicated their confidence (1–7). In Experiment 2A, Group 1 was instructed to imagine that the camera was “very far” from the surface plane, and Group 2 was instructed to imagine that the camera was “very near” to the surface plane. These instructions were intended to ensure that the imagined distance range could vary with the interpretations for each image. In Experiment 2B, Group 2 was presented with images that featured a large familiar object (airplane), and Group 3 was presented with images that featured a small familiar object (hornet). English translations of the original German instructions for all experiments are provided in the Methods S1 Supplemental Data.

### Quantification and statistical analysis

#### Statistical tests

All statistical tests were two-sided, with statistical significance defined as *p* < 0.05. Data distributions were checked for normality, and where this assumption was not met, non-parametric tests were used. One-sample binomial tests were used to determine whether mean categorization accuracies were significantly different from fixed values. Spearman rank correlations were used to determine the extent of agreement between free-response and multiple-choice material category frequencies (Experiment 1), and to compare results across Experiments 1 and 2. A novel statistic (Material-Scale Ambiguity, or MSA) was developed to measure the extent to which specific images caused distance-dependent category confusions. Wilcoxon signed rank tests were used to determine whether the observed MSA was significantly different from that produced by randomization of distance estimates (Experiment 1) or group membership (Experiment 2). A Mann–Whitney *U* test was used to compare mean confidence ratings across experiments. Detailed results, including specific values of statistical parameters, are reported in the Results section. Statistical calculations were made with the following software packages: Python v3.7.6 (Python Software Foundation, 2019), Jupyter Notebook v6.4.2 (Executable Books Community, 2020), NumPy v1.20.3 (Harris et al., 2020), Pandas v1.3.1 (Pandas Development Team, 2020), SciPy v1.6.2 (Virtanen et al., 2020), Scikit-learn v0.24.2 (Pedregosa et al., 2011), R v4.0.2 (R Core Team, 2018), rpy2 v3.4.5 (Gautier, 2021). Figures were created with the following software packages: Matplotlib v3.4.2 (Hunter, 2007), Seaborn v0.11.1 (Waskom, 2021), statannotations v0.4.3 (Charlier, 2021), spaCy v3.1.2 (Honnibal and Montani, 2021), TensorFlow v2.0.0 and TensorFlow Hub v0.10.0 (Abadi et al., 2015), MATLAB R2021a and MATLAB Engine API for Python (MATLAB, 2021). All code and data necessary to reproduce these results and figures is available in the public OSF repository listed in the key resources table.

#### Free-response transformation

Participants initially judged each image by providing a written description of the material, after which they selected a single material category from a list of options. To determine the extent of agreement between judgements of material in multiple-choice and free-response trials, the free-response data was manually reduced to single-word descriptions, which included all but one of the material categories available during the multiple-choice trials (“hair”), as well as the following 101 additional descriptors: aluminum, asphalt, basalt, bast, bone, brick, bronze, cake, canvas, cardboard, cellulose, cement, chalk, chalk stone, chocolate, cloud, coal, copper, coral, cord, cork, cotton, cotton candy, crystal, denim, detergent, dirt, dough, dust, screed, felt, fleece, flour, foam, foil, fungus, gel, gem, glue, gold, granite, graphite, grass, hay, honeycomb, iron, jade, jelly, jute, laminate, lead, lime, limestone, linen, meat, mirror, moss, mold, mud, none, nylon, oil, pearl, peat, photopaper, plaster, plexiglass, powder, pumice, putty, PVC, quartz, resin, rock, root, rubber, rust, salt, sandpaper, silicone, silk, silver, slate, slime, slom, smoke, soap, soil, sponge, steel, straw, styrofoam, sugar, sulfur, talcum, tar, velvet, vinyl, wheat, wool, hair

The free-response data was manually transformed using the following rules:

1.Corrected typos and spelling errors
2.Nouns only
  a.No adjectives (e.g., "scratched stone" = "stone")
  b.No noun adjuncts (e.g., "desert sand" = "sand")
3.Specific materials/stuff only (e.g., "landscape", "material", "don't know" = "none")
4.Translated to English

Resolve synonyms (e.g., "frozen water" = "ice")

The strong rank correlation between the frequency of common terms (*r*_*s*_ = 0.74, p < 0.001) indicated that multiple-choice responses capture the free-response ranking of material categories to a significant degree. Subsequent analyses of material judgements therefore focused on multiple-choice responses.

#### Distance estimate analysis

Distance estimates involved selecting a unit of measurement (micrometers, millimeters, centimeters, meters, or kilometers) before assigning a metric value (e.g., 8 cm, or 1 km). Although this task was designed to allow participants to quickly estimate very small or very large distances, the variance associated with each unit of measurement is unequal (e.g., distance estimates in kilometers will have a much larger variance than estimates in micrometers). When converted to a common unit (centimeters), therefore, the distribution of these values is extremely skewed (see Figure S2) and spans several orders of magnitude (*SD* = 106× 10^6^ cm). For this reason, subsequent analyses of distance estimates focused on the selected units of measurement, which represent judgements of viewing distance on an ordinal scale.

#### Relative frequencies

Multiple-choice responses for each image were first divided into two groups based on the distance unit selected with the material: responses selected with micrometer, millimeter, or centimeter defined the *near group*, while those selected with meter or kilometer defined the *far group*. Responses of the near group were then paired with responses of the far group, such that all pairwise permutations in both directions (near → far vs. far → near) were represented. This was done separately for each image (i.e., responses associated with different images were never paired). The frequency of each unique pair of responses represents the relative frequency of directional category confusions. The relative frequencies shown in Figure 3 are calculated from response pairs for all images. Material-Scale Ambiguity (MSA) is calculated from response pairs for individual images.

#### Exploratory analyses

In a separate pilot experiment, naive participants (4 women, 1 man; *M*_*age*_ = 24.4 years, *SD*_*age*_ = 4.1 years) rated five appearance attributes (atmospheric blue tint, blurriness, direct lighting, surface gloss, and surface slant) on a continuous scale for the original set of 87 images. A self-similarity (i.e., scale-invariance) metric was also calculated for each image (Brachmann and Redies, 2017). In order to assess whether this data might predict Material-Scale Ambiguity (MSA), a Support Vector Machine (SVM) classifier (Pedregosa et al., 2011) was trained on 1000 random splits of the data into training and test sets. The target variable for classification was defined as a median split of MSA calculated from data in Experiment 1. A separate SVM classifier (using a Radial Basis Function kernel) was trained for each set of training and test data. Optimal parameters (C and Gamma) for this kernel function were obtained separately for each model using a grid search method. The mean cross-validated classification accuracy across these 1000 models is 56%, which is only marginally better than the accuracy that would be expected from a model that only learns the distribution of the target variable. That is, if exactly half of the images produce MSA that is greater than the median MSA produced by the whole set of images, a classifier that guesses randomly would be expected to have an accuracy of 50%. In order to understand this failure, the mean rating of each appearance attribute was calculated separately for each image. The most frequent distance unit selected for each image was then identified and grouped according to the near and far distance groups defined in Experiment 1. Finally, the mean rating of each appearance attribute was calculated separately for each distance group. Figure S3 plots these distance-dependent attribute ratings, which reveals that four of the five attributes were not significantly different between distance groups. The one exception is surface slant, which was much more highly rated for images that depicted relatively large distances (Mann–Whitney *U =* 297, *p* < 0.001, *d* = 1.16).

Additionally, these participants rated the 26 material categories used in Experiment 1 along a continuous scale from “inorganic” (man-made) to “organic” (natural). We calculated the mean rating for each material category and created two groups from a median split of these values; “organic” categories had a mean rating above the median and “inorganic” categories had a mean rating below the median. We then calculated the frequency of these two category groups within the *near* and *far* distance groups defined in Experiment 1. Notably, “organic” material categories made up 76% of responses when participants estimated surfaces to be far from the camera, but only 45% when surfaces were estimated to be near to the camera (see Figure S3 in the Supplemental information). All data and code necessary to reproduce these analyses is available in the public OSF repository listed in the key resources table.

## Data Availability

• De-identified data for both experiments have been deposited at Open Science Framework and is publicly available as of the date of publication. The DOI is listed in the key resources table. • Analysis code for both experiments have been deposited at Open Science Framework and is publicly available as of the date of publication. The DOI is listed in the key resources table. • Stimulus images are provided except when copyright permission could not be obtained. Any additional information required to reanalyze the data reported in this paper is available from the lead contact upon request.

## Footnotes

Supplemental information can be found online at https://doi.org/10.1016/j.isci.2022.103970.
