# Supplementary material for: Scale ambiguities in material recognition
Source: iScience. 2022 Feb 22;25(3):103970. doi: 10.1016/j.isci.2022.103970 (PMC8914553; doi:10.1016/j.isci.2022.103970)
Supplement: Document S1. Figures S1–S4 and Table S1 [file mmc1.pdf]

iScience, Volume *25*

Supplemental information

Scale ambiguities in material recognition

Jacob R. Cheeseman, Roland W. Fleming, and Filipp Schmidt

Supplemental Information

Figure S1. Ground truth material category labels (a) and image file numbers (b), related to STAR Methods.

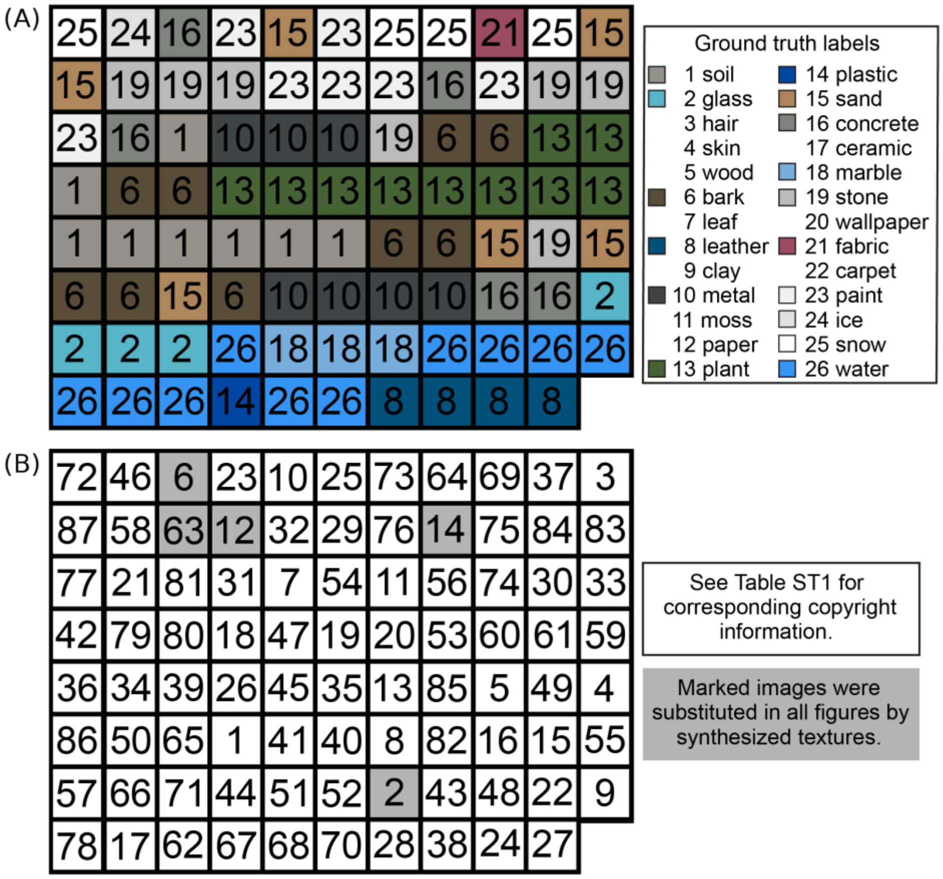

Figure S2. Log-scaled distribution of distance estimates (converted to centimeters), related to STAR Methods.

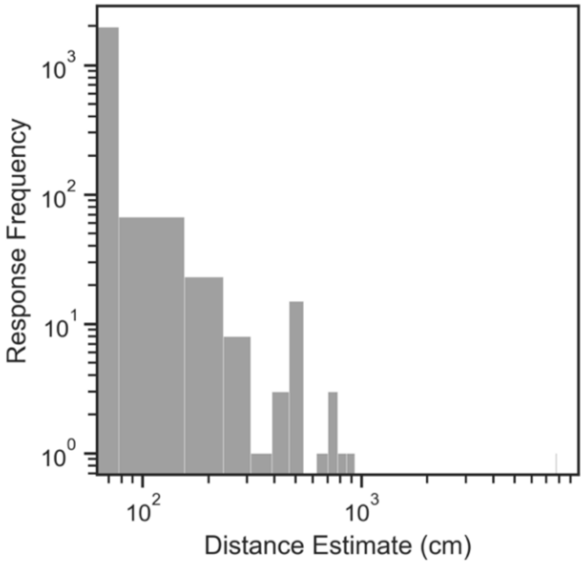

Figure S3. Mean ratings (across images) for each appearance attribute, related to STAR Methods.

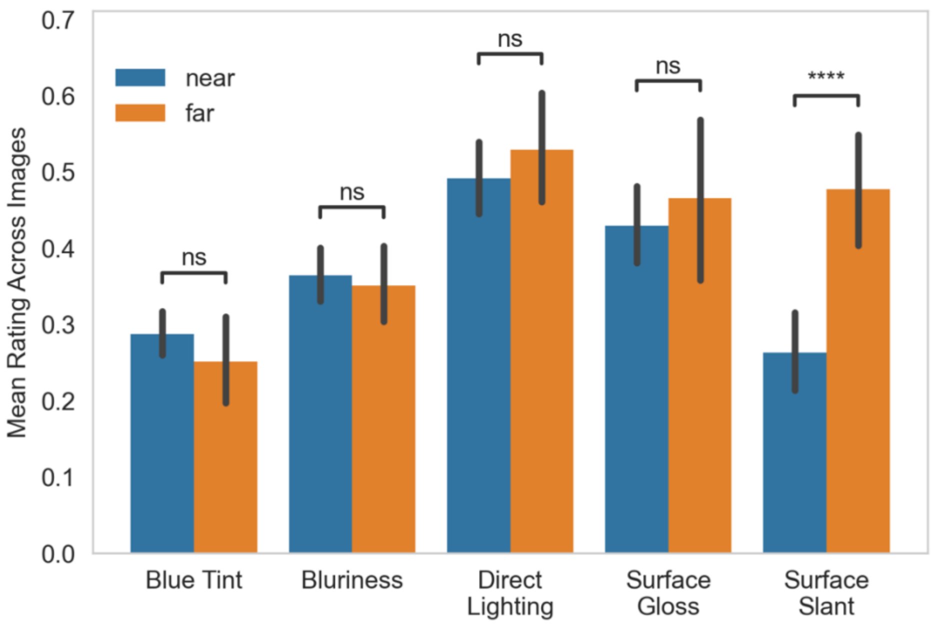

Figure S4. Distance-dependent frequency of categories rated as organic vs. inorganic, related to STAR Methods.

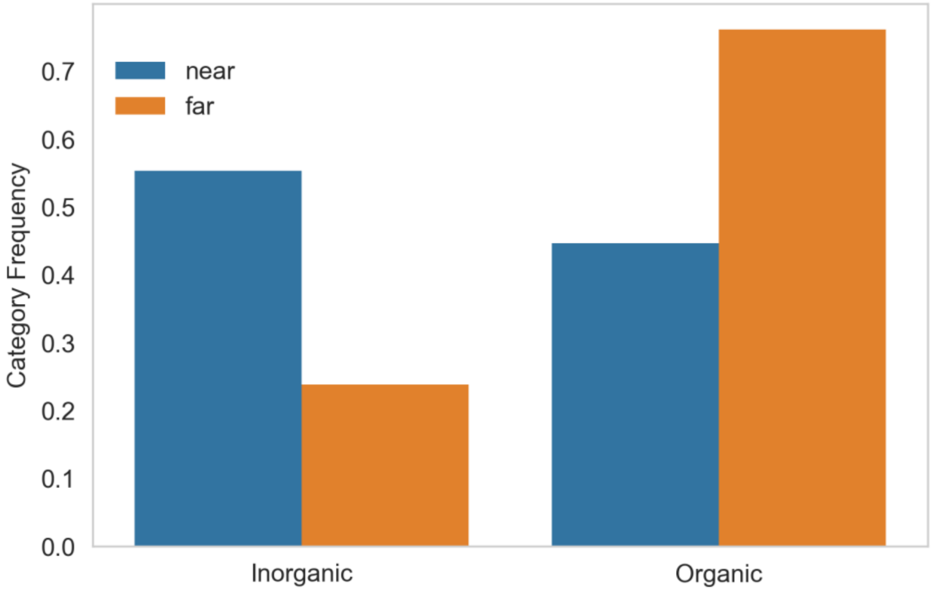

Table S1. Sources, copyright information and ground truth labels, related to STAR Methods.

No.	Copyright Information	Label
**1**	by unsplash.com/Lysander Yuen [reprinted under Unsplash License]	Bark
**2**	Print image substituted with synthesized texture [https://github.com/JCBrouwer/OptimalTextures]	Marble
**3**	by 123rf.com/Arina Zaiachin [reprinted with permission]	Sand
**4**	by 123rf.com/Juergen Schonnop [reprinted with permission]	Sand
**5**	by 123rf.com/Mikhail Kokhanchikov[reprinted with permission]	Sand
**6**	Print image substituted with synthesized texture [https://github.com/JCBrouwer/OptimalTextures]	Concrete
**7**	by freepick.com/LuqueStock [reprinted under Freepik-Lizenz]	Metal
**8**	by freepik.com [reprinted under Freepik-Lizenz]	Metal
**9**	by unsplash.com/Aidan Brown [reprinted under Unsplash License]	Water
**10**	by unsplash.com/Anchor Lee [reprinted under Unsplash License]	Sand
**11**	by freeImages.com/Brandon Blinkenberg [reprinted under freeimages content license]	Stone
**12**	Print image substituted with synthesized texture [https://github.com/JCBrouwer/OptimalTextures]	Stone
**13**	by pixabay.com/AnnaAr [reprinted under CC0 1.0]	Bark
**14**	Print image substituted with synthesized texture [https://github.com/JCBrouwer/OptimalTextures]	Concrete
**15**	by Wagner Treppenbau, wagner-treppenbau.de [reprinted with permission]	Concrete
**16**	by Wagner Treppenbau, wagner-treppenbau.de [reprinted with permission]	Concrete
**17**	by slon.pics [reprinted under slon.pics free license]	Water
**18**	by unsplash.com/Carlo Verso [reprinted under Unsplash License]	Plant
**19**	by unsplash.com/Carlo Verso [reprinted under Unsplash License]	Plant
**20**	by unsplash.com/Carlo Verso [reprinted under Unsplash License]	Plant
**21**	by freeImages.com/dlritter [reprinted under freeimages content license]	Concrete
**22**	by unsplash.com/Das Sasha [reprinted under Unsplash License]	Water
**23**	by depositphotos.com/Petkov [reprinted with permission]	Paint
**24**	by depositphotos.com/studioDG [reprinted with permission]	Leather
**25**	by depositphotos.com/Lunamarina [reprinted with permission]	Paint
**26**	by depositphotos.com/Sergieiev [reprinted with permission]	Soil
**27**	by depositphotos.com/mario7 [reprinted with permission]	Leather
**28**	by depositphotos.com/Homydesign [reprinted with permission]	Leather
**29**	by depositphotos.com/Mankukuku [reprinted with permission]	Paint
**30**	by depositphotos.com/Alexis84 [reprinted with permission]	Plant
**31**	by depositphotos.com/tuja66 [reprinted with permission]	Metal
**32**	by depositphotos.com/kues [reprinted with permission]	Paint
**33**	by depositphotos.com/Watman [reprinted with permission]	Plant
**34**	by depositphotos.com/alxbaev@gmail.com [reprinted with permission]	Soil
**35**	by depositphotos.com/wayne0216 [reprinted with permission]	Soil
**36**	by depositphotos.com/Watman [reprinted with permission]	Soil
**37**	by depositphotos.com/cristi180884 [reprinted with permission]	Snow
**38**	by depositphotos.com/Natalt [reprinted with permission]	Leather
**39**	by depositphotos.com/ekina1 [reprinted with permission]	Soil
**40**	by depositphotos.com/VitaliyPliushc [reprinted with permission]	Metal
**41**	by depositphotos.com/VitaliyPliushc [reprinted with permission]	Metal
**42**	by depositphotos.com/ViktoriaSapata [reprinted with permission]	Soil
**43**	by unsplash.com/Evelyn Fjord [reprinted under Unsplash License]	Water

**44**	by freeImages.com/Krzysztof Isbrandt<br>[reprinted under freeimages content license]	Water
**45**	by freeImages.com/Jose Mora [reprinted under freeimages content license]	Soil
**46**	by pixabay.com/TeroVesalainen [reprinted under CC0 1.0]	Ice
**47**	by unsplash.com/Ivan Bandura [reprinted under Unsplash License]	Plant
**48**	by unsplash.com/Jason Leem [reprinted under Unsplash License]	Water
**49**	by unsplash.com/Plenio [reprinted under Unsplash License]	Stone
**50**	by unsplash.com/Jude Infantini [reprinted under Unsplash License]	Bark
**51**	by bgfons.com [reprinted under CC BY-NC 4.0]	Marble
**52**	by 123freevectors.com[reprinted under 123freevectors License]	Marble
**53**	by unsplash.com/Matt Seymour [reprinted under Unsplash License]	Plant
**54**	by freeImages.com/Bjarne Henning Kvaale<br>[reprinted under freeimages content license]	Metal
**55**	by photos-public-domain.com [reprinted under CC0 1.0]	Glass
**56**	by unsplash.com/Nate Bell [reprinted under Unsplash License]	Bark
**57**	by freepick.com/bedneyimages [reprinted under Freepik-Lizenz]	Glass
**58**	by unsplash.com/Rainer Basten [reprinted under Unsplash License]	Stone
**59**	“Veld 2” 2016 by David Burdeny, courtesy of Kostuik Gallery<br>[reprinted with permission]	Plant
**60**	“Veld 6” 2016 by David Burdeny, courtesy of Kostuik Gallery<br>[reprinted with permission]	Plant
**61**	“Veld 1” 2016 by David Burdeny, courtesy of Kostuik Gallery<br>[reprinted with permission]	Plant
**62**	by jooinn.com [reprinted under CC0 1.0]	Water
63	Print image substituted with synthesized texture<br>[https://github.com/JCBrouwer/OptimalTextures]	Stone
**64**	by shutterstock.com/rattiya lamrod [reprinted with permission]	Snow
**65**	by shutterstock.com/SAHACHATZ [reprinted with permission]	Sand
**66**	by shutterstock.com/Fred Mastison [reprinted with permission]	Glass
**67**	by shutterstock.com /Quality Stock Arts [reprinted with permission]	Plastic
**68**	by shutterstock.com/Dudarev Mikhail [reprinted with permission]	Water
**69**	by shutterstock.com/Shulevskyy Volodymyr [reprinted with permission]	Fabric
**70**	by shutterstock.com/Dudarev Mikhail [reprinted with permission]	Water
**71**	by shutterstock.com/SoulQuess [reprinted with permission]	Glass
**72**	by freeImages.com/meral akbulut [reprinted under freeimages content license]	Snow
**73**	by freeImages.com/Michal Zacharzeweski<br>[reprinted under freeimages content license]	Snow
**74**	by unsplash.com/Steinar Engeland [reprinted under Unsplash License]	Bark
**75**	by pixabay.com/PellissierJP [reprinted under CC0 1.0]	Paint
**76**	by freeImages.com/Leo Celso [reprinted under freeimages content license]	Paint
**77**	by freeImages.com/Florian Florea [reprinted under freeimages content license]	Paint
**78**	by unsplash.com/Tim Johnson [reprinted under Unsplash License]	Water
**79**	by tonytextures.de [reprinted under tonytextures license]	Bark
**80**	by tonytextures.de [reprinted under tonytextures license]	Bark
**81**	by tonytextures.de [reprinted under tonytextures license]	Soil
**82**	by tonytextures.de [reprinted under tonytextures license]	Metal
**83**	by tonytextures.de [reprinted under tonytextures license]	Stone
**84**	by tonytextures.de [reprinted under tonytextures license]	Stone
**85**	by freeImages.com/Terry V. Haslett [reprinted under freeimages content license]	Bark
**86**	by freeImages.com/Rene Cerney [reprinted under freeimages content license]	Bark
**87**	by unsplash.com/Vanda Teixeira [reprinted under Unsplash License]	Sand
