# Supplementary material for: Scale ambiguities in material recognition
Source: iScience. 2022 Feb 22;25(3):103970. doi: 10.1016/j.isci.2022.103970 (PMC8914553; doi:10.1016/j.isci.2022.103970)
Supplement: Document S2. Methods S1 [file mmc2.pdf]

Experiment 1 instructions, related to STAR Methods

General instructions

Original German: „Lieber Teilnehmer, in diesem Experiment siehst Du Bilder verschiedener Materialien. Bitte schau Dir jedes Bild genau an und gib an, welches Material Du siehst. Insgesamt gibt es 87 Bilder. In einem ersten Durchgang sollst Du zu jedem Bild die Materialnamen, die Dir zutreffend erscheinen in ein Textfeld schreiben. In einem späteren, zweiten Durchgang werden Dir die selben Bilder noch einmal gezeigt und Du sollst den zutreffendsten Materialnamen aus einer Liste auswählen und anschließend angeben, wie zufrieden Du mit dieser Auswahl bist. In einem dritten Durchgang werden Dir die selben Bilder noch einmal gezeigt und Du sollst angeben wie weit Du glaubst, dass die Kamera im Moment der Aufnahme von dem Material entfernt war (z.B. 9 cm, 400 m, 12 km). Die Dauer des Experiments beträgt etwa 1,5 Stunden."

English translation: Dear participant, in this experiment you see pictures of different materials. Please take a close look at each picture and indicate which material you see. There are 87 images in total. In the first step you should write the material names for each picture that appear to be appropriate in a text field. In a later, second round, you will be shown the same pictures again and you should select the most appropriate material name from a list and then state how satisfied you are with this selection. In a third run you will be shown the same pictures again and you should indicate how far you think the camera was away from the material at the moment of the recording (e.g., 9 cm, 400 m, 12 km). The duration of the experiment is about 1.5 hours.

Free-response instructions

Original German: „Aus welchem Material besteht obiges Bild? Gib alle Materialnamen an, die Dir einfallen und das Material möglichst genau beschreiben. Wie überzeugt bist Du davon, dass das Bild wirklich dieses Material zeigt? (Wobei 1 bedeutet: sehr wenig überzeugt; 7 bedeutet: sehr überzeugt)"

English translation: What material is the above picture made of? Enter all material names that you can think of and that describe the material as precisely as possible. How convinced are you that the picture really shows this material? (Where 1 means: very little convinced; 7 means: very convinced)

Multiple-choice instructions

Original German: „Aus welchem Material besteht obiges Bild? Wähle aus dem Dropdown-Menü. ['Erde', 'Glas', 'Haar', 'Haut', 'Holz (Holz)', 'Holz (Rinde)', 'Laub', 'Leder', 'Lehm', 'Metall', 'Moos', 'Papier', 'Pflanzen', 'Plastik', 'Sand', 'Stein (Beton)', 'Stein (Keramik)', 'Stein (Marmor)', 'Stein (Stein)', 'Tapete', 'Textil (Stoff)', 'Textil (Teppich)', 'Wandfarbe', 'Wasser (Eis)', 'Wasser (Schnee)', 'Wasser (Wasser)'] Wie überzeugt bist Du davon, dass das Bild wirklich dieses Material zeigt? (Wobei 1 bedeutet: sehr wenig überzeugt; 7 bedeutet: sehr überzeugt)"

English translation: What material is the above picture made of? Select from the drop-down menu. ['soil', 'glass', 'hair', 'skin', 'wood', 'bark', 'leaves', 'leather', 'clay', 'metal', 'moss', 'paper', 'plants', 'plastic', 'sand', 'concrete', 'ceramic', 'marble', 'stone', 'wallpaper', 'fabric', 'carpet', 'paint', 'ice', 'snow', 'water'] How convinced are you that the picture really shows this material? (Where 1 means: very little convinced; 7 means: very convinced)

Distance-estimate instructions

Original German: „Was ist der Abstand zwischen der Kamera und der Oberfläche? Maßeinheit auswählen. ['Mikrometer', 'Millimeter', 'Zentimeter', 'Meter', 'Kilometer'] Welcher Wert in dieser Maßeinheit (z. B. 1,2 Zentimeter oder 2,5 Kilometer)?"

English translation: What is the distance between the camera and the surface? Select unit of measure. ['micrometer', 'millimeter', 'centimeter', 'meter', 'kilometer'] Which value in this unit of measurement (e.g. 1.2 centimeters or 2.5 kilometers)?

Experiment 2 instructions, related to STAR Methods

Experiment 2A instructions (far group)

Original German: „Lieber Teilnehmer, in diesem Experiment siehst Du Bilder verschiedener Materialien. Bitte schau Dir jedes Bild genau an und gib an, welches Material Du siehst. Insgesamt gibt es 87 Bilder. In einem ersten Durchgang sollst Du zu jedem Bild die Materialnamen, die Dir zutreffend erscheinen in ein Textfeld schreiben. In einem späteren, zweiten Durchgang werden Dir die selben Bilder noch einmal gezeigt und Du sollst den zutreffendsten Materialnamen aus einer Liste auswählen und anschliessend angeben, wie zufrieden Du mit dieser Auswahl bist. Berücksichtige, dass die Kamera im Moment der Aufnahme SEHR WEIT von dem Objekt/der Materialoberfläche entfernt war. Die Dauer des Experiments beträgt etwa 1 Stunde."

English translation: Dear participant, in this experiment you see pictures of different materials. Please take a close look at each picture and indicate which material you see. There are 87 images in total. In the first step you should write the material names for each picture that appear to be appropriate in a text field. In a later, second round, you will be shown the same pictures again and you should select the most appropriate material name from a list and then state how satisfied you are with this selection. Take into account that the camera was VERY FAR away from the object / material surface at the moment of recording. The duration of the experiment is about 1 hour.

Experiment 2A instructions (near group)

Original German: „Lieber Teilnehmer, in diesem Experiment siehst Du Bilder verschiedener Materialien. Bitte schau Dir jedes Bild genau an und gib an, welches Material Du siehst. Insgesamt gibt es 87 Bilder. In einem ersten Durchgang sollst Du zu jedem Bild die Materialnamen, die Dir zutreffend erscheinen in ein Textfeld schreiben. In einem späteren, zweiten Durchgang werden Dir die selben Bilder noch einmal gezeigt und Du sollst den zutreffendsten Materialnamen aus einer Liste auswählen und anschliessend angeben, wie zufrieden Du mit dieser Auswahl bist. Berücksichtige, dass die Kamera im Moment der Aufnahme SEHR NAH von dem Objekt/der Materialoberfläche entfernt war. Die Dauer des Experiments beträgt etwa 1 Stunde."

English translation: Dear participant, in this experiment you see pictures of different materials. Please take a close look at each picture and indicate which material you see. There are 87 images in total. In the first step you should write the material names for each picture that appear to be appropriate in a text field. In a later, second round, you will be shown the same pictures again and you should select the most appropriate material name from a list and then state how satisfied you are with this selection. Take into account that the camera was VERY NEAR to the object / material surface at the moment of recording. The duration of the experiment is about 1 hour.

Experiment 2B instructions

Original German: „Lieber Teilnehmer, in diesem Experiment siehst Du Bilder verschiedener Materialien. Bitte schau Dir jedes Bild genau an und gib an, welches Material Du siehst. Insgesamt gibt es 87 Bilder. In einem ersten Durchgang sollst Du zu jedem Bild die Materialnamen, die Dir zutreffend erscheinen in ein Textfeld schreiben. In einem späteren, zweiten Durchgang werden Dir die selben Bilder noch einmal gezeigt und Du sollst den zutreffendsten Materialnamen aus einer Liste auswählen und anschliessend angeben, wie zufrieden Du mit dieser Auswahl bist. Die Dauer des Experiments beträgt etwa 1 Stunde."

English translation: Dear participant, in this experiment you see pictures of different materials. Please take a close look at each picture and indicate which material you see. There are 87 images in total. In the first step you should write the material names for each picture that appear to be appropriate in a text field. In a later, second round, you will be shown the same pictures again and you should select the most appropriate material name from a list and then state how satisfied you are with this selection. The duration of the experiment is about 1 hour.
